# Clinical and functional evidence supporting pathogenicity of a novel APOA5 variant in familial chylomicronemia syndrome

**DOI:** 10.20945/2359-4292-2026-0101

**Published:** 2026-08-31

**Authors:** Johnayro Gutiérrez, Pablo Castaño, Claudia Monsalve, Nestor López, Fernando Rivera Toquica, Edwin Mora, Jessica Cristina Armijos, Juliana Lores, Henry Mauricio Arenas, Germán Camilo Giraldo, Diana Sánchez, Natalia Agudelo, Gregorio Fariña, Gabriela Berg, Juan Patricio Nogueira

**Affiliations:** 1 Servicio Endocrinología Clínica y Metabolismo, Universidad de Antioquia. Clínica Somer, Rionegro, Antioquia, Colombia; 2 Clínica Somer, Rionegro, Antioquia, Colombia; 3 Universidad Pontificia Bolivariana. Clínica las Américas AUNA, Medellín, Colombia; 4 Universidad Pontificia Bolivariana. Clínica Somer, Rionegro, Antioquia, Colombia; 5 Clínica Los Rosales S.A, Pereira, Colombia; 6 Universidad de Caldas, Caldas, Colombia; 7 Clínica San Rafael, Pereira, Risaralda, Colombia; 8 Unidad de Medicina Genómica y Genética, Clínica Imbanaco, Cali, Colombia; 9 Departamento de Ciencias Básicas, Facultad de Ciencias de la Salud, Pontificia Universidad Javeriana Cali, Colombia; 10 Universidad Tecnológica de Pereira, Department of Endocrinology, Comfamiliar Risaralda, Pereira, Risaralda; 11 Pureza de Corazón IPS. Manizales, Caldas, Colombia; 12 Mrg medical operations (Ex Brazil). PTC Therapeutics, Bogotá, Colombia; 13 PTC Therapeutics, Bogotá, Bogotá, Colombia; 14 Laboratorio de Lípidos y Aterosclerosis, Departamento de Bioquímica Clínica, Facultad de Farmacia y Bioquímica, Universidad de Buenos Aires, Buenos Aires, Argentina; 15 Centro de Investigación en Endocrinologia, Nutricion y Metabolismo, Facultad de Ciencias de la Salud, Universidad Nacional de Formosa, Formosa, Argentina

**Keywords:** Familial chylomicronemia syndrome, hypertriglyceridemia, *APOA5*, pancreatitis, dyslipidemia

## Abstract

**Objective:**

This study aimed to evaluate the clinical and functional impact of a novel apolipoprotein A5 (ApoA5) variant by assessing lipoprotein lipase (LPL) activity.

**Subjects and methods:**

Demographic and clinical data, including blood lipid levels and body mass index, were retrospectively collected from an endocrinology clinic registry. Ten individuals with familial chylomicronemia syndrome (FCS) carrying a novel *APOA5* variant were included. Whole-exome sequencing was performed using the latest generation DNB-SEQ400 platform. LPL activity was measured in post-heparin plasma using a radiometric assay. Statistical analysis: Categorical variables were summarized as frequencies and percentages, and continuous variables as mean ± standard deviation or median (range), as appropriate. Group comparisons were performed using the Mann-Whitney U test or chi-square test. Analyses were conducted using the Statistical Package for the Social Sciences software (v. 25.0). A p-value < 0.05 was considered statistically significant.

**Results:**

We report ten cases of FCS with a homozygous missense variant of uncertain significance in *APOA5*: c.694T>C; p.(Ser232Pro), located in exon 3. In six patients assessed for LPL activity, levels remained consistently below 20% compared to normotriglyceridemic controls. The addition of exogenous serum containing *APOA5* restored LPL activity to above 20% in all cases, indicating functional rescue.

**Conclusion:**

Measurement of LPL activity demonstrated the functional impact of the APOA5 c.694T>C; p.(Ser232Pro) variant. These findings support reclassification of the variant as likely pathogenic and enable differentiation from multifactorial chylomicronemia syndrome.

## INTRODUCTION

Apolipoprotein A5 (*ApoA5*) is a critical regulator of plasma triglyceride (TG) levels (^1^). It modulates hepatic synthesis of very low-density lipoprotein (^2^), enhances lipoprotein lipase–mediated triglyceride clearance (^3^), and promotes hepatic uptake of TG-rich lipoprotein remnants via the low-density lipoprotein (LDL) receptor family (^4^). *ApoA5* enhances LPL activity through interactions with heparan sulfate proteoglycans (^5^) and glycosylphosphatidylinositol-anchored high-density lipoprotein-binding protein 1 (*Gpihbp1*) on endothelial cell surfaces (^6^). Biallelic loss-of-function (LOF) variants in *APOA5* underlie a rare condition with an estimated prevalence of 1–10 per million (^7^) and represent a known cause of familial chylomicronemia syndrome (FCS) (^8^,^9^). Even severely truncated *APOA5* variants are synthesized and secreted (^10^); however, premature truncation can disrupt key functional domains, leading to protein misfolding. In homozygous or compound heterozygous states, these alterations result in a significant reduction in *APOA5* mass and function (^11^,^12^). Only 38% of *APOA5* variants are classified as pathogenic or likely pathogenic (^13^), most of which are missense mutations. When genetic testing yields negative or inconclusive results, measurement of LPL activity can confirm the diagnosis of FCS, particularly when activity is reduced below 20–25% of the normalmedian (^14^,^15^). In this study, we describe the clinical and genetic characteristics of patients with severe hypertriglyceridemia carrying *APOA5* variants and assess their pathogenicity by measuring LPL activity in ten affected individuals.

## SUBJECTS AND METHODS

Ten patients (3 men, 7 women; mean age: 42 ± 3.2 years) with strong clinical suspicion for FCS were included. Serum TG, total cholesterol, low-density lipoprotein cholesterol, high-density lipoprotein cholesterol, and apolipoprotein B were measured using commercial enzymatic assays on a Mindray BS360E autoanalyzer (Mindray, China). Body mass index and the Moulin score were calculated (^16^). Secondary causes of hypertriglyceridemia were excluded.

### DNA preparation and sequencing

Peripheral blood samples were collected from all patients, and whole-exome sequencing was performed using the latest-generation DNB-SEQ400 platform. Genes associated with FCS (e.g., *LPL*, *APOC2*, *APOA5*, *GPIHBP1*, and *LMF1*) were analyzed. High-quality reads were aligned to the human reference genome (GRCh37/hg19). The analysis focused on variants in exonic and splice-site regions (minimum 20 bp), as well as small insertions and deletions (copy number variants). Variant classification followed the American College of Medical Genetics and Genomics (ACMG) guidelines (^13^), using the ClinGen framework (clinicalgenome.org). Databases consulted included Clinical Variation database, Human Gene Mutation Database, Leiden Open Variation Database, Database of Single Nucleotide Polymorphisms, Genome Aggregation Database, and in-house resources. The identified variant exhibited extremely low allelic frequency in the general population (0.000002502 in gnomAD v. 4.1), consistent with a pathogenic allele in a recessive inheritance model. *In silico* prediction tools (REVEL, BayesDel) classified the variant as deleterious.

### LPL activity

To assess LPL activity, heparin was administered intravenously at a dose of 60 IU/kg body weight. Ten minutes after injection, blood samples were collected from the contralateral arm into ice-chilled tubes. Post-heparin plasma was obtained by centrifugation at 1500 × g for 10 minutes at 4 °C and stored at -70 °C until analysis. LPL activity was measured in post-heparin plasma from six patients. A standardized LPL activity assay was developed in our laboratory based on the Nilsson-Ehle method (^17^,^18^). This method has previously been applied in patients with FCS, in whom a median LPL activity of 33.3 mU/L (95% confidence interval, 28.5–41.3) was established for normotriglyceridemic (NTG) subjects (^19^). The intra-assay coefficient of variation was 5.1%, whereas the inter-assay coefficient of variation was 13.2%. Given the complexity of the assay, these values are considered acceptable. The calculated limit of detection was 0.144 mIU, and the limit of quantification was 1.83 mIU. A value of 6.6 mIU corresponds to 20% of normal activity. Therefore, LPL activity below this threshold is supportive of a diagnosis of FCS.

A functional assay was also performed. Two substrates were prepared, one of which included 10% v/v normolipemic human serum, heat-inactivated at 56 °C for 30 minutes and used as a source of exogenous cofactors, including ApoC-II and ApoA5. The *in vitro* assay was used to evaluate potential cofactor deficiency (^18^).

### *In silico* evaluation of APOA5-LPL: *GPIHBP1* interactions

Because no crystal structure is available for human *APOA5*, its structure was obtained from the AlphaFold Protein Structure Database (ID: Q6Q788) and used as a template. Structural modeling of the identified variant was performed using SWISS-MODEL, followed by energy minimization with SwissPDB Viewer (^20^). Energy-minimized models of both the native and mutant *ApoA5* proteins, along with the LPL–*Gpihbp1* complex (PDB ID: 6E7K), were uploaded to the HDOCK server, which calculates docking scores; lower scores indicate more favorable binding interactions (^21^).

### Statistical analysis

Categorical variables were summarized using frequencies and percentages, while continuous variables were described using mean, standard deviation, median, and range (minimum–maximum). Results are presented as mean values with their corresponding standard errors. Comparisons between groups were performed using the Mann-Whitney U test or the Chi-square test, as appropriate. Statistical analyses were conducted using the Statistical Package for the Social Sciences software (v. 25.0). A *p*-value < 0.05 was considered statistically significant.

## RESULTS

**Table 1** summarizes the clinical and biochemical characteristics of the patients. All had a Moulin score ≥10 and a history of recurrent pancreatitis. The patients were unrelated and from different regions of Colombia. All carried the same homozygous variant in the *APOA5* gene: c.694T>C; p.(Ser232Pro). This missense variant, located in exon 3, results from a T-to-C substitution at nucleotide position 694, leading to a serine-to-proline substitution at amino acid position 232. To date, it has been classified as a variant of uncertain significance (^9^).

**Table 1.** Demographic, clinical, biochemical data, and LPL activity levels before and after addition of activator serum in the ten cases

Case	Sex	Age (years)	TG	HDL-c	LDL-c	ApoB	BMI (kg/m^2^)	MS	PPDM	AP	AP episodes and age at first episode	LPL (mIU)	Activity of median NTG (%)	LPL with AS (mIU)	Activity of median NTG with AS (%)
			mg/dL												
1	F	54	2748±345	19±3	31±8.3	75±7.9	24.6	12	No	Yes	1, 48 Y	6.35	19.05	15.80	47.40
2	M	68	5530±416	11±2.1	34±9.2	35.9±8.4	29.0	12	No	Yes	3, 27 Y	2.37	7.11	11.59	34.78
3	F	36	5128±398	18±3.1	U	U	24.3	12	No	Yes	5, 31 Y	5.97	17.91	19.75	59.26
4	M	64	5808±472	9.0±1.3	12±7.8	93±11.5	26.5	10	No	No		6.19	18.08	14.59	43.76
5	F	64	1664±178	13±4.2	40±9.1	98±12.4	20.1	13	No	Yes	1, 40 Y	3	9.0	8.67	26.00
6	F	65	1600±225	39±4.6	47±11.3	47±7.5	27.47	10	No	No		9.47	9.47	10.39	31.16
7	F	84	1105±248	30.4±4	89±12.4	47±9.3	30.4	10	U	No		NP	NP	NP	NP
8	F	52	4200±459	31±3.1	U	U	23.9	12	No	Yes	3, 30 Y	NP	NP	NP	NP
9	F	50	3638±298	19.3±2.4	100±14.6	U	27.1	10	U	Yes	1, 36 Y	NP	NP	NP	NP
10	F	54	1000±192	20.9±2.6	U	U	29.0	11	Yes	Yes	8, 45 Y	NP	NP	NP	NP

Gray rows correspond to patients who underwent lipoprotein lipase activity assessment.

AP: Acute pancreatitis; ApoB: apolipoprotein B; AS: activator serum; BMI: body mass index; F: female; HDL-C: high-density lipoprotein cholesterol; LDL-C: low-density lipoprotein cholesterol; LPL: lipoprotein lipase; M: male; MS: Moulin score; NP: not performed; NTG: normotriglyceridemic controls; PPDM: post-pancreatitis diabetes mellitus; TG: triglycerides (mg/dL); U: unknown; Y: years.

AS: activator serum; LPL: lipoprotein lipase; NTG: normotriglyceridemic controls.

### LPL activity and functional assay

**Figure 1.**
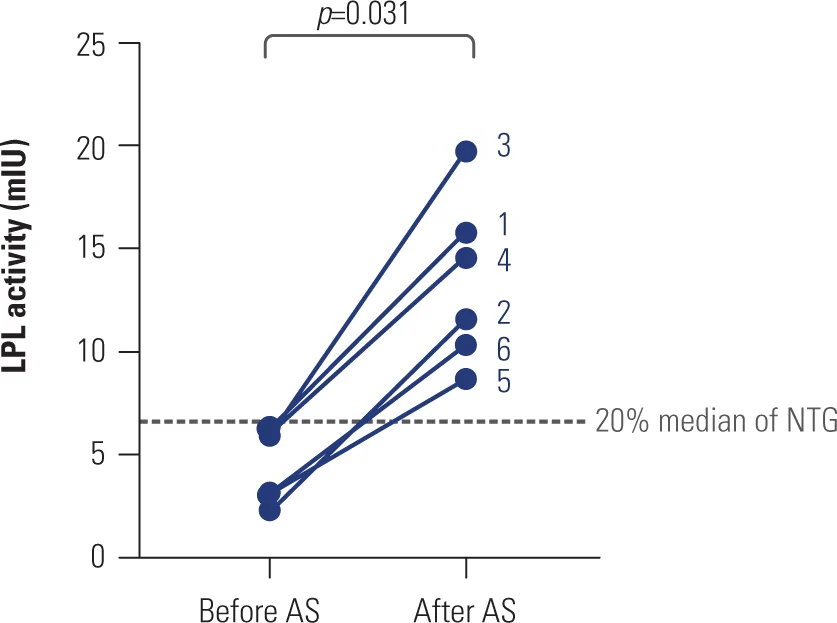
LPL activity levels before and after the addition of activator serum. The dotted line indicates 20% of the median LPL activity observed in NTG controls. Statistical comparisons were performed using the Wilcoxon signed-rank test.

LPL activity for six patients are shown in **Figure 1**, along with the results of the *in vitro* functional assay. In all cases, LPL activity was below 20% of that observed in NTG controls. The *in vitro* assay, which included the addition of normal serum as a source of key cofactors (i.e., *ApoC-II* *and ApoA5*), demonstrated restoration of LPL activity to normal levels in all cases. These findings suggest that LPL function was preserved in the presence of functional *ApoA5* (**Table 1**). In four patients, samples could not be collected in time for shipment to Argentina for LPL activity analysis. No differences were observed between patients with and without LPL activity measurements in terms of diabetes status, history of pancreatitis, or lipid parameters, confirming the severity of the novel *APOA5* variant.

## DISCUSSION

This study describes ten patients carrying a novel homozygous *APOA5* variant, initially classified as a variant of uncertain significance. In patients with high clinical suspicion scores and markedly reduced LPL activity, these findings provide support for reclassifying this variant as likely pathogenic. *ApoA5* contains several functional domains: a signal peptide, an N-terminal hydrophilic domain, a lipid droplet-binding domain, a positively charged *Gpihbp1*-interacting domain, and a C-terminal hydrophobic (lipid-binding domain) (^11^). Molecular analysis identified the homozygous variant NM_001371904.1: c.694T>C; p.(Ser232Pro), located in exon 3. This substitution impairs the interaction between *ApoA5* and *Gpihbp1*. The introduction of a proline residue induces local conformational rigidity, altering the spatial orientation of residues critical for *GPIHBP1* recognition. In addition, substitution of a polar serine with proline may affect binding affinity and kinetics of the *ApoA5-Gplhbp1* interaction, and its role in facilitating LPL-mediated TG lipolysis (^22^).

At least 118 definite or likely disease-causing variants in *APOA5* have been reported in the literature and databases, and are associated with FCS, hypertriglyceridemia, and atherosclerotic disease. Even minor alterations in the protein may impair its lipid-binding properties. The remaining three pathogenic LOF variants in *APOA5* could be associated with substantial functional impairment (^22^). The variant identified (*APOA5*: c.694T>C; p.Ser232Pro) likely disrupts Gpihbp1 interaction, similar to other pathogenic variants such as p.Ser232_Leu235del and p.Leu253Pro, resulting in impaired very low-density lipoprotein secretion and reduced LPL activity (^12^). No evidence of a dominant-negative effect has been reported for p.Ser232Pro. In previously reported cases (^9^), the variant showed a recessive pattern of inheritance, and heterozygous carriers exhibited normal or mildly elevated TG levels. These observations are consistent with a LOF mechanism, likely driven by impairment of the ApoA5-Gplhbp1 interaction.

Although the findings provide strong evidence for a deleterious effect of the *APOA5* c.694T>C (p.Ser232Pro) variant, the current classification as likely pathogenic is based on ACMG/AMP criteria that do not yet meet the threshold for pathogenicity. The study shows markedly reduced LPL activity (<20% of normal), with restoration upon addition of exogenous ApoA5, supporting a LOF mechanism. However, ACMG/AMP guidelines require either multiple independent functional studies or well-validated assays to support reclassification beyond this level (^13^).

The available genetic and functional evidence supports a LOF effect and disease association. Nonetheless, the data meet ACMG/AMP criteria for “likely pathogenic” classification. Additional independent functional studies, segregation analyses, and replication in other cohorts are necessary to support full classification as “pathogenic.” Following ACMG/AMP guidelines for variant interpretation (^13^) (ClinGen framework updates), the variant met criteria PM3 (strong), PM2 (supporting), and PP3 (supporting), and was therefore classified as “likely pathogenic.” Overall, p.Ser232Pro behaves similarly to other structurally disruptive *APOA5* variants, with pathogenicity consistent with a recessive LOF mechanism (^21^). The clinical and functional data support the pathogenicity of this variant.

LPL activity below 20% is strongly associated with a diagnosis of FCS and with the exclusion of multifactorial chylomicronemia syndrome (^23^,^24^), in combination with the Moulin score (^16^), and North American FCS scores (^25^). In our cohort, LPL activity was consistently below 20% of NTG controls, supporting the pathogenicity of this variant. Genetic testing fails to provide definitive results in a substantial proportion of FCS patients, even when the Moulin and North American FCS scores scores strongly suggest a high likelihood of disease (^16^,^25^). The APPROACH study reported that 21% of participants lacked identifiable genetic variants but were included based on their clinical presentation and markedly reduced LPL activity (^14^). LPL activity below 20% is strongly associated with FCS diagnosis and supports the exclusion of multifactorial chylomicronemia syndrome (^23^,^24^). In our cohort, LPL activity was consistently below 20% of NTG controls, supporting the pathogenicity of this variant. Although LPL activity testing is complex and not widely available, it is valuable in cases with inconclusive genetic results or unclear differentiation between FCS and multifactorial chylomicronemia syndrome. We recently reported a case of a pregnant patient with severe hypertriglyceridemia (6900 mg/dL) and a history of pancreatitis (^24^). Genetic testing identified a heterozygous *APOA5* variant (c.111delC; p.Asp37Glufs*20), classified as likely pathogenic. LPL activity was below 20% (9.6%), increasing to 31.2% after addition of activator serum, supporting a diagnosis of FCS and reclassification of the variant.

In conclusion, our findings showed that rare variants in *APOA5* play a critical role in the pathophysiology of FCS. In this study, we documented reduced LPL activity, supporting the diagnosis and demonstrating the functional impact of the *APOA5* variant. We propose a founder effect for this novel homozygous *APOA5* variant, which has also been reported in a prior case classified as a variant of uncertain significance. Novel *APOA5* variants are likely to be reclassified as pathogenic and LPL activity data continue to accumulate.

## Data Availability

datasets related to this article will be available upon request to the corresponding author.
